# Prevalence of metabolic syndrome and components in rural, semi-urban and urban areas in the littoral region in Cameroon: impact of physical activity

**DOI:** 10.1186/s41043-023-00415-0

**Published:** 2023-09-11

**Authors:** Nadine Carole Bilog, Jerson Mekoulou Ndongo, Elysée Claude Bika Lele, Wiliam Richard Guessogo, Peguy Brice Assomo-Ndemba, Ahmadou, Noel Babayana Etaga, Yves Julien Mbama Biloa, Josiane Gertrude Bwegne Ngasse Bindi, Abdou Temfemo, Samuel Honoré Mandengue, Jessica Guyot, Caroline Dupré, Nathalie Barth, Bienvenu Bongue, Laurent Serge Etoundi Ngoa, Clarisse Noel Ayina Ayina

**Affiliations:** 1 Department of Biology of Animal Organisms, Faculty of Science, University of Douala, Douala, Cameroun; 2 Exercise and Sport Physiology Unit, Faculty of Science, University of Douala, Douala, Cameroon; 3 National Institute for Youth and Sports Yaounde, University of Yaounde I, Yaounde, Cameroon; 4 Faculty of Medicine and Pharmaceutical Sciences, University of Douala, Douala, Cameroon; 5 Department of Physiology, Faculty of Medicine and Biomedical Sciences, University of Yaounde I, Yaounde, Cameroon; 6 Mines Saint-Etienne, INSERM, SAINBIOSE U1059, 42023 Saint-Étienne, France; 7 Department of Animal Science, Higher Teacher’s Training College, University of Yaoundé I, Yaounde, Cameroon

**Keywords:** Physical activity, Metabolic syndrome, Urban, Semi-urban, And rural areas, Littoral, Cameroon

## Abstract

**Background:**

Living areas in developing countries impact seriously lifestyle by modifying energy consumption and energy expenditure. Thus, urbanization is associated with less practice of physical activity (PA), a leading cause of metabolic syndrome (MetS) which prevalence vary in African countries. The present study aimed to assess the effect of PA on MetS according to urbanization level in the littoral region, Cameroon.

**Methods:**

A cross-sectional study was conducted in three geographical settings (urban, semi-urban, and rural) in the littoral region in Cameroon. A total of 879 participants were included (urban: 372, semi-urban: 195 and rural: 312). MetS was defined according to the International Federation of Diabetes 2009. The level of PA was assessed using the Global Physical Activity questionnaire.

**Results:**

Low level of PA was (*P* < 0.0001) reported in urban (54.5%), semi-urban (28.7%) and rural (16.9%) and high level in rural area (77.9%). The prevalence of MetS was higher in urban areas (37.2%), then rural (36.8%) and finally semi-urban (25.9%). Hyperglycemia (*p* = 0.0110), low HDL-c (*p* < 0.0001) and high triglyceridemia (*p* = 0.0068) were most prevalent in urban residents. Participants with low level of PA were at risk of MetS (OR: 1.751, 95% CI 1.335–2.731, *p* = 0.001), hyperglycemia (OR: 1.909, 95% CI 1.335–2.731, *p* = 0.0004) abdominal obesity(OR: 2.007, 95% CI 1.389–2.900, *p* = 0.0002*)*, low HDL-c (OR: 1.539, 95% CI 1.088–2.179, *p* = 0.014) and those with moderate level of PA were protected against high blood pressure(OR: 0.452, 95% CI 0.298–0.686, *p* = 0.0002) and compared to those with high level of PA. Urban dwellers were at the risk of MetS compared to rural residents (OR: 1.708, 95% CI. 1.277–2.285, *p* = 0.003) and protected against high blood pressure (OR:0.314, 95% CI 0.212–0.466,* p* < 0.0001), abdominal obesity (OR: 0.570, 95% CI 0.409–0.794,* p* = 0.0009), and low HDL-c (OR: 0.725, 95% CI 0.534–0.983,* p* = 0.038) compared to rural residents.

**Conclusions:**

MetS was more prevalent in urban dwellers and was associated with a low level of PA.

## Background

According to the world health organization (WHO), there is an increase in urbanization. The world’s population living in urban areas is 54%, and that proportion is expected to reach 66% in 2050. Projections show that urbanization combined with the overall growth of the world’s population could add another 2.5 billion people to urban populations by 2050, with close to 90% of the increase concentrated in Asia and Africa [1]. Most of the expected urban growth will take place in developing countries in Asia and Africa [1].

Living areas in African countries are commonly associated with an increased prevalence of non-communicable diseases and cardiovascular risk factors with high incidence in urban region [2]. Urbanization has a profound impact on the physical environment leading to air pollution, urban heat islands, and other effects, which are known to have an impact on metabolic health [3]**.** Living area is also associated with changes in nutrition comportments such as increased consumption of energy-rich foods and an increase in physical inactivity which is the leading cause of non-communicable diseases such as obesity, hypertension, and diabetes which are most prevalent in urban areas [4]. Metabolic syndrome (MetS) is defined as a cluster of abnormalities including abdominal obesity, hyperglycemia, hypertension, and dyslipidemia present in the same individual [5, 6].

The prevalence of MetS has reached epidemic proportions worldwide and varies between populations, race, gender, socio-economic status [7], and geographical settings [8]. MetS is a reality in Africa, the prevalence of the MetS varie between different populations in Africa [9], and it prevalence ranges from 0 to 50% or even higher depending on the population, areas (rural and urban) and the used criteria [10].

Sub-Saharan African countries which are the least urbanized in the world are facing one of the fastest rates of urbanization in the world, perceptible through rapid demographic and epidemiologic transitions [11]. MetS is also associated with physical inactivity [12] which is recognized as a global pandemic, responsible for more than 5 million deaths per year.

Physical inactivity is one of the primary targets to reduce non-communicable diseases [13–15]. In 2018, WHO updated the global recommendations on physical activity for the health of 2010, based on the latest available science, including sedentary behavior, in the urgent need to achieve a global reduction of 15% of physical inactivity in order to promote health in 2030 [16]. Physical activity (PA) contributes to preventing and managing non-communicable diseases and cluster components of MetS, and it lack is a major cause of chronic diseases [17, 18]. Decreased levels of PA are negatively correlated with health, the environment, economic development, community well-being, and quality of life. Insufficiently active people have a 20–30% increased risk of death compared to sufficiently active people [18]. WHO reported an important decrease in the practice of PA and a proportion of adults aged 18 and over was more concerned in 2016 [16, 18]. This decrease in PA is related to the level of urbanization, in high-income countries, 26% of men and 35% of women were insufficiently physically active compared to 12% of men and 24% of women in low-income countries [18]. Low or decreasing PA levels often correspond with a high or rising gross national product [18]. PA and its impacts on non-communicable diseases are related to socio-economic grounds and the living areas [19]. The association between PA, geographical settings, and MetS is worrying in developing countries. Hence, the present study aimed to determine prevalence of MetS according to living areas and the impact of PA in the littoral region in Cameroon.

## Methods

### Study design and study population

This was a cross-sectional and prospective study conducted in the littoral region of Cameroon. The littoral region is one of the ten administrative regions of Cameroon. The littoral region is an important economic region in which the economic capital is found. The study was conducted in the urban, semi-urban, and rural areas of the region.

Geographical settings were defined according to the BUCREP criteria [20]. The urban area is characterized by a high population density, mainly composed of civil servants, businessmen, and students, with important infrastructures, and a high level of urbanization. In a rural area, there is no agglomeration of population, the populations for the most part draw their income from agriculture, fishing and breeding. The semi-urban zone is located halfway between urban and rural areas [20].

### Sample

The study ran from April to August 2021. Participants were randomly recruited and were of both genders aged 18 years and more residing in the study areas during the study period. Pregnant and lactating women, persons with cardiometabolic diseases, those on medication, and with physical disabilities were not included in our study. The study minimum sample was calculated using the Lorentz formulas with a prevalence of 8.4% of obesity reported by Tachang et al. [21] and the minimum was 120 participants. A total of 879 participants were recruited, and thus constituted, urban: 372, semi-urban: 195 and rural: 312 participants.

### Ethics approval

The study was approved by the Institutional Ethics Committee for Human Health Research of the University of Douala (No CE-UDO/07/2020/T), and by the regional delegation of the Ministry of Public Health. The study was conducted in accordance with the guidelines of the Helsinki Declaration of 1975, as revised in 2008. Also, research authorizations granted to hospital administration staff were obtained. Written informed consents were obtained from all. Data were collected anonymously and were confidential.

### Socio-demographic and behavioral informations

A questionnaire developed from the World Health Organization (WHO) STEPS manual for surveillance of risk factors of NCDs and adapted to the study context was used to collect socio-demographic information (age, level of education, marital status, medication diagnostic cardiometabolic disease, etc.) information on habits related to healthy living, alcohol intake and smoking (answering by “yes” or “no” to the question) in particular; and information about the medical history of study participants. The Alcohol Use Disorders Identification Test (AUDIT) was used to assess the participant’s alcohol consumption.

### Measurements

#### Anthropometric

Weight and body composition were measured using a bioelectric impedance meter Terraillon Wellness Coach (USA). Height was measured using a measuring tape. The body mass index (BMI) was calculated to assess the degree of obesity of each participant according to the Quetelet formula as the weight (Kg) divided by the square of the height (m^2^). The participants were classified according to their BMI as follows: normal weight (BMI < 25), overweight (BMI ≥ 25–29.9,) and obese (BMI > 30 kg/m^2^). Waist circumference (WC) was measured with an inelastic tape between the lower edge of the costal arch and the iliac bone’s upper crest in a standing position with an accuracy of 0.5 cm [22] and waist-to-height ratio (WHtR) was calculated as WC divided by the height.

#### Blood pressure and heart rate

Systolic blood pressure (SBP), diastolic blood pressure (DBP), and heart rate (HR) were taken using an electronic blood pressure monitor (JERISON, China) placed on the subject’s left arm in a sitting position. The first measurement was taken after a 10 min rest in a sitting position and was followed by another measurement after 5 min intervals, the average of the two measurements was used to assess the presence or absence of high blood pressure**.**

#### Physical activity

Levels of PA were determined based on the Global Physical Activity Questionnaire (GPAQ) analysis guide developed by WHO [23]. This questionnaire comprises 16 questions grouped to capture PA undertaken in different behavioral domains; these are work, transport, and leisure or recreation time during a typical week. GPAQ collects information on the practice of physical activities (frequency, duration, and intensity of activities) and on sedentary behavior. The questionnaire takes into account activities at work, during transport, and leisure activities. The results were scored and participants were classified as having low, moderate and high levels of PA.

### Biochemistry

For each fasting subject (12–14 h of fasting), blood glucose was measured between 8 and 10 am using a MyStar Extra glucometer (SANOFI, China). Blood samples were taken from the ulnar vein, and a volume of 10 ml was collected and conserved in EDTA tubes by venepuncture in the hand of each participant. The sera were obtained by blood centrifugation at 4000 rpm for 20 min in a Techmel & Techmel (USA) centrifuge and then placed in cryotubes, and aliquots were frozen at -20 °C for further biochemical analyses.

The levels of total cholesterol (TC), HDL-C, and triglycerides (TG) were determined using a UVmini 1240 spectrophotometer (SHIMADZU) according to the Biorex kit material (respectively, Cholesterol CHO-Rev 01 of 1271/2008, Triglycerides BXC0271 and HDL CHO-Rev 01 of 1271/2008) [24, 25]**.** Low-density lipoprotein (LDL) was calculated using the Friedewald et al. [26] formula if the triglycerides are less than 400 mg/dl (4.6 mmol/l). LDL-c was calculated by subtracting HDL-c and VLDL from total cholesterol. The serum standards used for calibration were provided by the manufacturer.

The insulin resistance was assessed by calculating the Homeostatic Model Assessment Insulin resistance (HOMA-IR) using the following

$${\text{HOMA}} - {\text{ IR }} = \frac{{\left[ {{\text{C}} - {\text{peptide}}} \right]{ } \times {\text{fasting glucose}}}}{22.5}$$

The C-peptide blood concentration was determined by ELISA (enzyme-linked immunosorbent assay) using the Mercodia C-peptide Ultrasensitive ELISA test (Mercodia AB Sylveniusgatan 8A SE-754 50 Uppsala, Sweden). The special Mercodia C-peptide ELISA kit is calibrated using the International Reference Reagent for C-peptide, IRR C-peptide 84/510 [27]. The HOMA-IR was evaluated in 600 individuals, 200 per area which were submitted to dosage of C-peptide.

### Metabolic syndrome criteria

Harmonized definition of MetS by the International Diabetes Federation of 2009 [6] was used whose:

- central obesity that was defined by a waist circumference ≥ 94 cm in men and ≥ 80 cm in women,
- High fasting glucose level ≥ 100 mg/dL (5.6 mmol/L);
- hypertriglyceridemia-serum triglyceride level ≥ 150 mg/dL (1.7 mmol/L);
- Low HDL cholesterol-serum; HDL cholesterol < 40 mg/dL (1.0 mmol/L) in men and < 50 mg/dL (1.3 mmol/L) in females);
- High blood pressure [systolic blood pressure (SBP) ≥ 130 mmHg and/or diastolic blood pressure (DBP) ≥ 85 mmHg.

Participants with 3 or more of the 5 MetS components were considered to have MetS.

### Statistical analysis

Statistical analysis was performed with the Statistical Package for Social Sciences (SPSS) software, Version 21.0 (SPSS, Inc. Chicago, U.S.A. IBM Corp.). Results of sociodemographic, behavioral information, ponderal status, MetS and its components and level of PA were expressed as proportions (%) for quantitative variables and means ± standard deviation (SD), the distribution pattern of variables was checked. The Chi-2 test was performed to compare unpaired proportions and Student’s *t *test on unpaired series was performed to compare quantitative variables between urban, semi-urban and rural areas. Stepwise multivariate analysis was performed to examine the association between the level of PA, MetS and its cluster components, also between geographical settings, MetS and its components. The significance was set with a *p*-value < 0.05.

## Results

The distribution of participants by living area varied significantly with respect to sociodemographic information (Table 1). More than 50% of males were living in rural areas, while most of the females (51.1%) were living in urban areas (*p* < 0.0001). A large majority of participants living in urban area were under 35 years and those over 35 years old were in the rural area (*p* < 0.0001). More than half (70.9%) of participants having completed primary studies were living in a rural area, while 55.2% of those having completed secondary studies were living in urban areas (Table 1). Also, participants smoking and drinking alcohol were more in a rural area (51.4%, *p* = 0.006 and 39.5%, *p* = 0.0002, respectively) It should be noted that 77.9% of those having a high level of physical activity (PA) were living in rural areas (*p* < 0.0001). In contrast, 51.3% and 54.5% of those having either moderate or low levels of PA were living in urban areas. A low level of PA was significantly reported in females compared to males in urban (87.4%, *p* < 0.001), semi-urban (70.1%, *p* < 0.01) and rural (64.3%, *p* < 0.01).

**Table 1:** Sociodemographic characterization of the participants

Parameters	Categories	Overall %	Urban %	Semi-urban %	Rural %	*p*-value
Gender	Male	38.2	28.6	20.5	50.9	< 0.0001
	Female	61.8	51.0	23.0	26.0	

Parameters	Categories	Overall %	Urban %			Semi-urban %			Rural %			*p* value
			All	Female	Male	All	Female	Male	All	Female	Male	
Age (years)	< 35	66.0	56.4	62.9	37.1	20.0	67.2	32.8	23.6	69.7	30.3**	< 0.0001
	> 35	34.0	15.1	53.2	46.8	26.4	57.8	42.2	58.5	50.5	49.5**	
Ponderal status	Normal	63.5	46.3	54.0	46.0**	20.4	62.5	437.5	33.3	60.1	39.9	0.001
	Overweight	22.4	41.1	75.9	24.1**	24.9	74.3	25.7	34.0	71.7	28.3	
	Obesity	14.1	26.6	67.6	32.4	25.8	60.0	40.0	47.6	63.3	36.7	
Level of study	None	3.2	10.7	33.3	66.7***	46.4	76.9	23.1	42.9	33.3	66.7**	< 0.0001
	Primary	16.1	6.4	55.6***	44.4	22.7	66.7	33.3	70.9	61.0	39.0**	
	Secondary	68.7	55.2	78.4***	21.6	19.4	64.1	35.9	25.4	41.8	58.2**	
	University	12.1	25.5	37.0	63.0***	30.2	56.3	43.7	44.3	25.5	74.5	
Marital status	Single	63.9	59.0	78.2***	21.8	18.7	64.2	35.8	22.3	35.2	64.8***	< 0.0001
	Cohabitation	5.8	2.0	0.0	100***	37.3	78.9	21.1	60.8	48.4	51.6***	
	Divorced	1.3	18.2	50.0	50.0	36.4	50.0	50.0	45.5	40.0	60.0***	
	Married	24.6	13.9	40.0	60.0***	26.9	55.2	44.8	59.3	45.3	54.7***	
	Widowed	4.4	20.5	62.5***	37.5	20.5	100	100	59.0	95.7	4.3***	
Toxicology behaviors	Smoking	3.9	34.3	8.3	91.7***	14.3	0	100**	51.4	5.6	94.4**	0.006
	Alcohol intake	58.5	36.6	76.1	23.9	23.9	65.9	34.1	39.5	46.3	53.7	0.0002
Level of PA	High	25.7	11.9	7.4	92.6***	10.2	34.8**	65.2	77.9	40.3	59.7**	< 0.0001
	Moderate	36.2	51.3	71.2**	28.8	23.6	65.3**	34.7	25.2	42.5	57.5**	
	Low	38.1	54.5	87.4***	12.6	28.7	70.1**	29.9	16.9	64.3	35.7**	

PA: Physical activity; p values are for comparison between males and females; *p < 0.05, **p < 0.01, ***p < 0.0001

Anthropometric, physiological and lipid profile data by living areas are depicted in Table 2. Participants from the urban area had the lowest mean weight values compared to their counterparts from semi-urban areas and rural areas. Likely, the same patterns were observed for WC (*p* < 0.0001) and WhtR (*p* < 0.0001). Body water was significantly higher in participants from rural areas while those from semi-urban areas had the highest values of body fat and bone mass. SBP DBP and HR also varied significantly between areas, with, for instance, highest HR values in urban areas compared to those from semi-urban and rural settings. Regarding lipid profile, glycemia and triglyceridemia were both higher in individuals from rural areas, while TC, HDL and LDL were highest in those from the urban settings, and the differences were all statistically significant (Table 2).

**Table 2:** Anthropometric, physiological and lipidemia profile of participants

	Total	Urban	Semi-urban	Rural
Age (years)	31 ± 15	23 ± 10 α***	35 ± 11 β***	39 ± 14 ɤ**
Height (m)	1.68 ± 0.56	1.65 ± 0.08	1.65 ± 0.09	1.72 ± 0.93
Weight (kg)	68.1 ± 21.2	65.4 ± 27.4 α**	68.8 ± 14.6	70.9 ± 14.9
BMI (Kg/m^2^)	24.7 ± 7.4	23.9 ± 9.3 α*	25.3 ± 5.4	30.1 ± 5.8
WC (cm)	80.6 ± 12.6	76.19 ± 10.82 α***	82.56 ± 13.1 β***	84.7 ± 12.7
WhtR	0.96 ± 0.04	0.46 ± .0.07 α***	0.50 ± 0.08 β***	0.51 ± .0.09
Body water (%)	55.0 ± 7.2	54.3 ± 6.8 α**	54.3 ± 8.7	56.51 ± 7.58 ɤ**
Body fat (%)	24.5 ± 12.4	23.4 ± 9.4	25.5 ± 10.4 β**	25.1 ± 16.1
Muscular mass (%)	36.3 ± 12.6	37.3 ± 7.3	34.8 ± 7.8 β**	36.1 ± 18.6
Bone mass (%)	3.1 ± 0.6	3.1 ± 2.2 α**	3.2 ± 3.7	2.8 ± 0.5
SBP (mmHg)	121 ± 18	117 ± 14 α***	125 ± 17 β***	125 ± 21
DBP (mmHg)	74 ± 13	70 ± 11 α***	76 ± 11 β***	78 ± 15 ɤ*
HR (bpm)	78 ± 14	82 ± 14 α***	78 ± 13 β**	74 ± 13 ɤ**
Glycemia (mg. dL^−1^)	106.7 ± 19.3	106.9 ± 14.2 α**	106.2 ± 22.2 β***	106.9 ± 22.5 ɤ***
T-Chol (g.L^−1^)	2.13 ± 0.52	2.23 ± 0.54	1.87 ± 0.36 β***	2.19 ± 0.54 ɤ***
HDL-c (g.L^−1^)	0.43 ± 0.13	0.47 ± 0.13 α***	0.36 ± 0.13 β***	0.42 ± 0.11 ɤ**
LDL-c (g.L^−1^)	1.30 ± 0.52	1.35 ± 0.54	1.13 ± 0.37 β***	1.35 ± 0.55 ɤ***
Triglyceridemia (g.L^−1^)	2.01 ± 0.4	2.03 ± 0.41 α**	1.80 ± 0.27 β***	2.11 ± 0.39 ɤ***
Homa-IR	5.9 ± 2.9	2.7 ± 1.3	3.0 ± 1.6	2.7 ± 1.3
MetS	2628 ± 120	1097 ± 65 α***	1335 ± 81 β*	5258 ± 266 ɤ***

SBP: Systolic Blood Pressure; DBP:Diastolic Blood Pressure; WC: Waist Circumference, BMI:Body Mass Index; WhtR: Waist To Hip Ratio; HR: Heart Rate; T-Chol: Total Cholesterol; HDL-C: High-Density Lipoprotein Cholesterol; LDL-c: Ligh-Density Lipoprotein Cholesterol, METS: Equivalent Metabolic Task; Homa-IR: Homeostatic Model Assessment Insulin Resistance, α: significant difference Rural–Urban; β: significant difference Semi-Urban-Urban; ɤ: significant difference Rural-Semi-urban; *p < 0.05, **p < 0.01, ***p < 0.0001

**Fig. 1:**
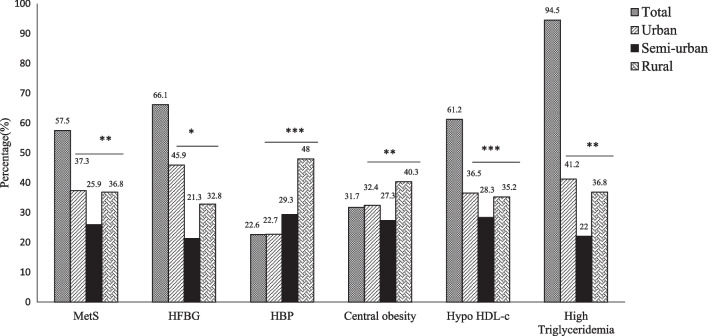
MetS and components according to living areas. HBP: high blood pressure, HFBG: High levels of fasting blood glucose, Hypo HDL-c: Low levels of blood HDL-c, MetS: Metabolic syndrome, Data are expressed as percentage, * < 0.05; ** < 0.01; *** < 0.001 as determined by Chi^2^test

Overall MetS was diagnosed in 57.7% of the participants and was more prevalent in female (*p* < 0.0001) in the overall population and each geographic setting (urban, semi-urban, and rural). The prevalence of MetS components varied geographically significantly (*p* < 0.05) with highest values found in participants from urban areas (37.2%) and rural (36.8%). Then, HFBG (*p* = 0.0110), low HDL-c (*p* < 0.0001) and high triglyceridemia (*p* = 0.0068) were most prevalent in participants from urban settings (Fig. 1). In contrast, HBP (*p* < 0.0001) and abdominal obesity (*p* = 0.0002) were most found among individuals from rural settings.

**Fig. 2:**
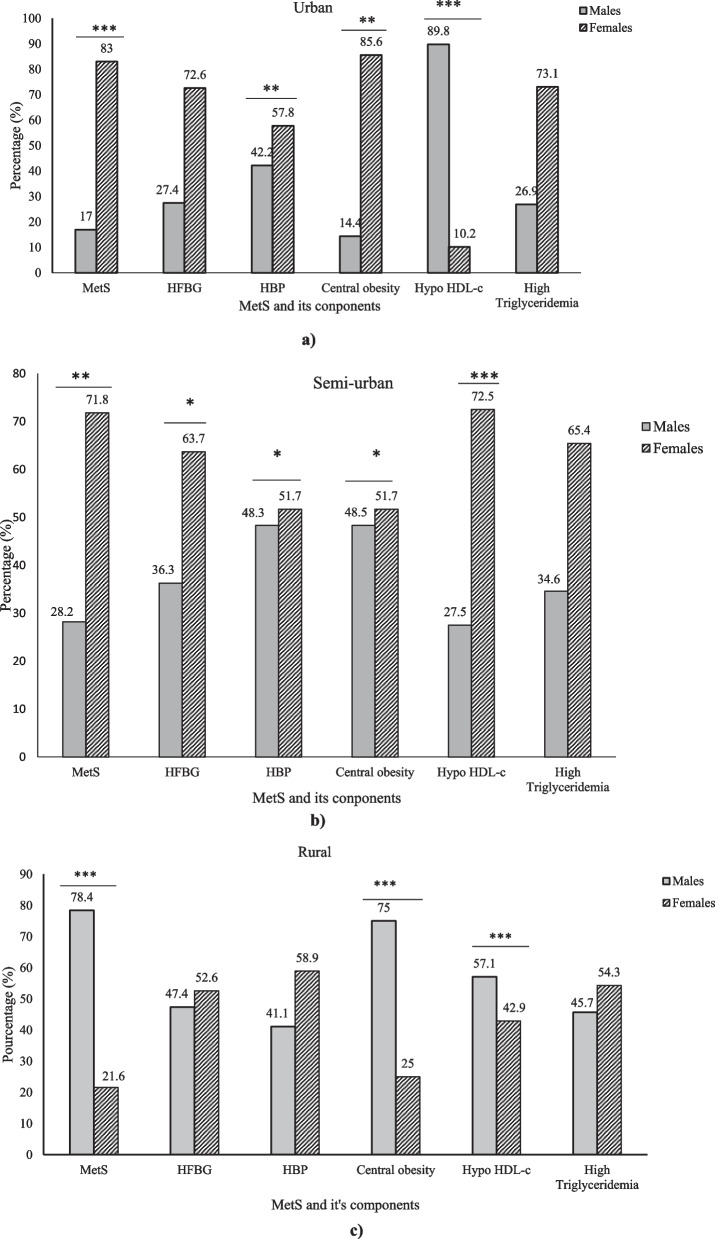
MetS and components according to gender in every living areas. HBP: high blood pressure, HFBG: High levels of fasting blood glucose, Hypo HDL-c: Low levels of blood HDL-c, MetS: Metabolic syndrome, Data are expressed as percentage, * < 0.05; ** < 0.01; *** < 0.001 as determined by Chi^2^test **a** MetS and components according to gender in urban region, **b** MetS and components according to gender in semi-urban region, **c** MetS and components according to gender in rural region

According to gender, the more prominent MetS components were HFBG, HBP, Abdominal obesity and Low HDL-c. Therefore, it has been noticed that HBP was more prevalent in male in overall population (52%, p < 0.0001) and superior in female in urban (87.8%, *p* < 0.01) and semi-urban (51.7%, *p* < 0.05). Abdominal obesity and low HDL-c were high in female in a study population (81.7%, *p* < 0.0001 and 73.4%, *p* < 0.0001, respectively) in each study site, in urban (85.6%, *p* < 0.01*;* 89.8%, *p* < 0.001 respectively*)*, semi-urban (51.7%, *p* < 0.05*;* 72.5%. *p* < 0.001, respectively) and rural areas (75.0%, *p* < 0.001*;* 57.1%, *p* < 0.001, respectively). HFBG was high in female compared to male in semi-urban region (63.7%, *p* < 0.05) (Fig. 2).

**Fig. 3:**
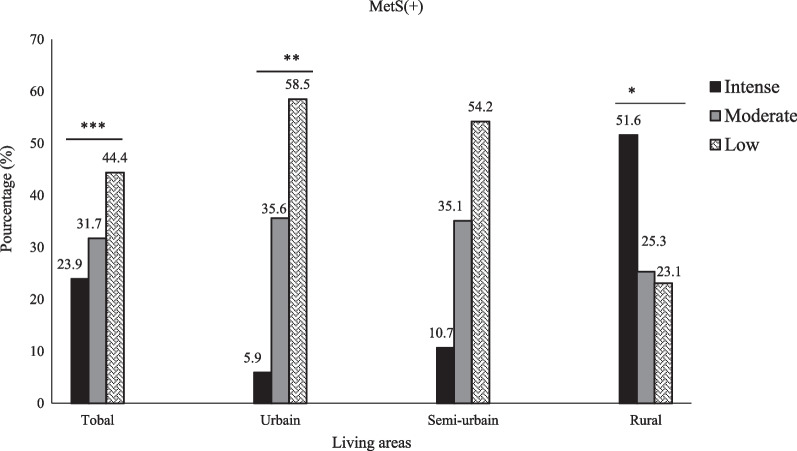
Prevalence of MetS according to level of physical activities and living areas MetS(+): Metabolic syndrome present

An inverted relation between MetS burden and level of PA was noted. This syndrome was more prevalent (*p* < 0.0001*)* in participants with a low level of PA (44.36%) compared to their counterparts with PA classified as moderate (31.68%) and high (23.96%). Curiously, we did not consistently find this pattern upon stratification by living areas. This pattern was noticed in urban areas only where the prevalence of MetS was 58.5% in participants having low, compared to those with moderate and high PA levels. In rural areas, MetS prevalence was significantly (*p* = 0.01) highest in individuals with a high level of PA, while no statistically significant difference was found in semi-urban settings (Fig. 3).

Multivariate analysis show that compared to participants with high level of PA those with low level were significantly at risk of MetS (OR: 1.751, 95% CI 1.335–2.731, *p* = 0.001) HFBG (OR: 1.909, 95% CI 1.335–2.731, *p* = 0.0004*)* abdominal obesity(OR: 2.007, 95% CI 1.389–2.900, *p* = 0.0002*)*, low HDL-c (OR: 1.539, 95% CI 1.088–2.179, *p* = 0.014) and those with moderate level of PA were protected against HBP(OR: 0.452, 95% CI 0.298–0.686, *p* = 0.0002) and compared to those with high level of PA. According to geographical setting urban dwellers had 1.708 great risk of MetS compared to rural residents and were also at risk of HFBG (OR:1.611, 95% CI 1.170–2.219, *p* = 0.003) and protected against HBP (OR:0.314, 95% CI 0.212–0.466,* p* < 0.0001), Abdominal obesity (OR:0.570, 95% CI 0.409–0.794,* p* = 0.0009), and Low HDL-c (OR:0.725, 95% CI 0.534–0.983,* p* = 0.038) compared to rural residents. However, semi-urban participants were at risk of Low HDL-c (OR: 2.371, 95% CI 1.573–3.573,* p* < 0.0001) compared to rural site. In addition, participants from urban areas compared to those from rural areas had a 70% reduction in the risk of high triglyceridemia (OR: 0.300, 95% CI 0.135–0.664,* p* = 0.003) (Table 3).

**Table 3:** Association between level of PA, geographical setting, MetS and its components

		MetS		HFBG		HBP		Abdominal Obesity		Low HDL-c		High triglyceridemia	
		OR (95%CI)	P	OR (95%CI)	P	OR (95%CI)	P	OR (95%CI)	P	OR (95%CI)	P	OR (95%CI)	P
PA	High	1		1		1		1		1		1	
	Moderate	0.879 (0.624–1.234)	0.458	1.274 (0.898–1.808)	0.174	0.452 (0.298–0.686)	0.0002	0.951 (0.644–1.406)	0.802	1.194 (0.845–1.686)	0.315	0.503 (0.208–1.218)	0.127
	Low	1.751 (1.238–2477)	0.001	1.909 (1.335–2.731)	0.0004	0.786 (0.537–1.149)	0.2133	2.007 (1.389–2.900)	0.0002	1.539 (1.088–2.179)	0.014	0.455 (0.191–1.083)	0.751
Locality	Rural	1		1		1		1		1		1	
	Semi-urban	1.387 (0.953–2.02)	0.08	0.544 (0.775–1.623)	0.544	0.967 (0.654–1.429)	0.866	1.140 (0.788–1.650)	0.485	2.371 (1.573–3.573	< 0.0001	0.487 (0.189–1.256)	0.136
	Urban	1.708 (1.277–2.285)	0.0003	1.611 (1.170–2.219)	0.003	0.314 (0.212–0.466)	< 0.0001	0.570 (0.409–0.794)	0.0009	0.725 (0.534–0.983)	0.038	0.300 (0.135–0.664)	0.003

MetS: Metabolic syndrome, OR: Odds ratio, PA: physical activity, HFBG: High fasting glucose, HBP: high blood pressure, HDL-c: high-density lipoprotein cholesterol

## Discussion

The present study aimed to determine the impact of PA on the occurrence of MetS in living areas. According to the criteria defined by the IDF consensus in 2009, the prevalence of MetS shows high prevalence in urban participants, followed by rural areas and semi-urban areas.

Other studies highlighted that MetS is emerging alarmingly in urban populations in low-income countries. These results are in accordance with those reported by others researchers who found high prevalence of MetS in urban dwellers compared to rural. In a study conducted in Benin by Ntandou et al.[29] on MetS and its components between urban, semi-urban and rural dwellers using the National Cholesterol Education Program-Adult Treatment Panel III (NCEP-ATP III) criteria noticed high prevalence in urban (11%) followed by semi-urban (6.4%) and rural population (4.1%). In Cameroon, a study focused on PA and the MetS among adults in the rural and urban regions by Assah et al. [30] using the NCEP-ATP III criteria reported high prevalence of MetS in urban dwellers (17.7%) compared to rural (3.5%). Even, Ntentie et al. [30] in a cross-sectional study also in Cameroon between urban, less urbanized, and rural areas reported a low prevalence of MetS in a rural area (7%) and high in less urbanized (17.4%) and urbanized areas (12.7%). Thanikachalam et al. [3] also assessed the impact of built environmental changes on the prevalence of MetS in a rapidly urbanizing population in South India and reported a high prevalence of MetS in urban (44.6%) and semi-urban communities (35.2%) compared to rural (31.8%).

The prevalence of MetS in the present study and some of its prominent cluster components were high in women. This result is in accordance with those of other researchers who established that MetS is more prevalent in women [30–33]. In a recent systematic review and meta-analysis on the prevalence of MetS in sub-Saharan Africa, Faijer-Westerink et al. [34] highlighted that the prevalence of MetS was highest in urban areas precisely in women. Even Ntandou et al. [28] in Benin found high levels of MetS, abdominal obesity, and low HDL-c in women than in men.

Moreover, our results don’t corroborate some of those reported by Ayina et al. [35] in a study in one administrative subdivision of our study region where they studied the urban–rural difference of MetS components. They reported high levels of total cholesterol, high triglyceridemia, waist circumference, and hyperglycemia in urban dwellers [35]. But according to the gender, some of their results were different from ours where they noticed high prevalence of total cholesterol, waist circumference, and low levels of HDL-c in urban men urban dwellers compared to females [35].

However, it has been observed the persistence of MetS central obesity and HFBG in the rural environment despite high and moderate level PA noticed (Fig. 1 & Table 1) and rural participants were at risk of HBP, abdominal obesity, and low HDL-c and protected against HFBG (Table 3). According to Popkin [36], this positive obesity gradient noticed in females in each study site can be ascribed to the westernization a nutrition transition process that is ongoing in developing countries. This result will be related to age and toxicological behaviors. More than half of the participants of the rural site (58.5%) were over 35 years old and were smoking (51.4%) and consuming alcohol (39.5%) than those living in urban and semi-urban sites (Table 1). Also, nearly half (47.6%) of the rural population was obese (Table 1).

It is well known that age [37], alcohol [38] and smoking cigarettes [39] constitute important risk factors for MetS. Thus, the regular practice of PA can’t be a real means of primary prevention of MetS. On the physiological aspect, cigarette smoking stimulates the sympathetic nervous system with increase glycogen and lipid catabolism [40] and also increases blood cortisol and growth hormone [39]. Tobacco smoking also elevates plasma cortisol concentration and aggravated insulin resistance can cause localization of visceral fat mass and increase abdominal obesity. This is in accordance with a study of Fezeu et al. [41] who noticed in Cameroon that urbanization is characterized by an increase in body mass index in the rural area and obesity which is considered as a major factor of the MetS [42]. Also, one of the first changes appears in cities before reaching less urbanized areas and exposing the people to obesity and other nutrition-related chronic diseases [36].

We observed a positive relationship between low PA and MetS in the overall study population and in urban and semi-urban. Also, low PA was associated with HFBG, abdominal obesity, and low HDL-c (Table 3). In the present study participants living in rural areas were more active with high (77.9%) and moderate (25.2%) prevalence of PA compared to those in urban and semi-urban (Table 1). Other studies have highlighted those rural dwellers were more active with high level PA [29, 43, 44]. Assah et al. [29] in a study on the urban–rural difference in energy expenditure, PA, and MetS; noticed that a low level of PA was strongly independently associated with a high prevalence of MetS in Cameroonians urban dwellers compared to rural residents.

Other studies reported a high level of PA in rural dwellers compared to the urban and low prevalence of MetS and some of its components [30, 45, 46]. Even, Ntentie et al. [30] have established that low level of PA was strongly associated with the occurrence of MetS. Moreover, rural–urban difference in PA with high level of rural residents has been linked to non-communicable diseases. This difference reflects the differences prevalence of in non-communicable diseases between rural–urban populations in Africa with high prevalence in the urban population [47–49]. However, it should be highlight that the level of PA in urbanized environments is inherent to socio-economic conditions, in particular the built environment where it has been shown its impact throughout the life. In a recent study conducted in South Africa Wayas et al. [50] found that perceived walkability among adolescents from low-income neighborhoods was worse than that from high-income neighborhoods, although the association with PA and ‘BMI is not clear.

Herein, many studies showed the evidence that PA practiced at a moderate and high level is a powerful tool for the primary prevention of metabolic disease and that it exerts its protective effects by improving the metabolic phenotype of non-skeletal muscle tissues, including the liver, vasculature, adipose tissue and pancreas [51]. In a physiologic way, PA exerts a positive effect by reducing components of MetS. There is evidence that high level of PA and MetS parameters has an inverse association, and persons with high level of PA have lower risks of developing MetS. Low levels of PA are associated with increased adiposity and decreased blood pressure, LDL-c, and higher HDL cholesterol [52]. PA improves lipid profile by increasing HDL concentration and decreasing LDL and triglyceride concentrations [53]. Previously several studies have determined the association between the level of PA and the prevalence of Mets [54–56]. The mechanism of high PA on MetS might be related to an important reduction of the level of inflammation. PA practiced at moderate and high levels is associated with a better profile of inflammatory factors and adipocytokines that lower MetS. Also, another possible physiological justification is the stimulation of the secretion of pro-inflammatory cytokines by a high level of PA. Regular practice of PA also improves body composition, dyslipidemia, and endothelial function also increases anti-inflammatory cytokines, decreases body fat, and decreases adhesion molecules expression [57–59].

## Conclusions

This study highlights the effect of living areas on the Mets, with a high prevalence of MetS in urban areas. This high prevalence of Mets and some of its components in urban areas are linked to a significant decrease in the practice of PA at high intensities by the inhabitants of these geographical settings. This emphasizes the use of PA as a non-pharmacological intervention for the primary prevention of MetS and its components in developing countries. Thus, the regular practice of PA in urban areas becomes an unavoidable necessity to fight against non-communicable diseases associated with urbanization. However, the practice of PA should be associated with the observation of hygienic behaviors, without smoking and alcoholism.

## Data Availability

Data can be shared upon contact with the correspondence author.

## Abbreviations

PA: Physical activity
SBP: Systolic blood pressure
DBP: Diastolic blood pressure
WC: Waist circumference
BMI: Body mass index
WhtR: Waist to hip ratio
T-Chol: Total cholesterol
HDL-c: High-density lipoprotein cholesterol
LDL-c: Low-density-lipoprotein-cholesterol
MET équivalent: Metabolic equivalent

## References

[1] United Nations. World urbanization prospects: the 2014 revision (high-lights). New York: United Nations Department of Economic and Social, 2014; Contract No.:ESA/P/WP/224.

[2] Vorster HH (2002). The emergence of cardiovascular disease during urbanization of Africans. Public Health Nutr.

[3] Thanikachalam M, Lane K, Thanikachalam S (2021). Abstract 12096: satellite-based urbanization measures are independent predictors of metabolic syndrome. Circ.

[4] World Health Organization. Global health risks: mortality and disease burden attributable to certain major risks. 2009. Geneva.

[5] Alberti KG, Zimmet P, Shaw J (2006). Metabolic syndrome—a new world-wide definition. A consensus statement from the international diabetes federation. Diabet Med.

[6] Alberti KG, Eckel RH, Grundy SM, Zimmet PZ, Cleeman JI, Donato KA, Fruchart JC, James WP, Loria CM, Smith SC (2009). Harmonizing the metabolic syndrome: a joint interim statement of the international diabetes federation task force on epidemiology and prevention; national heart, lung, and blood institute; American heart association; world heart federation; international atherosclerosis society; and international association for the study of obesity. Circulation.

[7] Gotto AM, Blackburn GL, Dailey GE, Garber AJ, Grundy SM, Sobel BE, Weir MR (2006). The metabolic syndrome: a call to action. Coron Artery Dis.

[8] Xu S, Ming J, Yang C, Gao B, Wan Y, Xing Y, Zhang L, Ji Q (2014). Urban, semi-urban and rural difference in the prevalence of metabolic syndrome in Shaanxi province, northwestern China: a population-based survey. BMC Public Health.

[9] Okafor CI (2012). The metabolic syndrome in Africa: current trends. Indian J Endocrinol Metab.

[10] Fezeu L, Balkau B, Kengne AP, Sobngwi E, Mbanya JC (2007). Metabolic syndrome in a sub-Saharan African setting: central obesity may be the key determinant. Atherosclerosis.

[11] Maire B, Delpeuch F (2014). Food and nutritional transition, and cities, in developing countries. Cah Agric.

[12] Rao DP, Orpana H, Krewski D (2016). Physical activity and non-movement behaviors: their independent and combined associations with metabolic syndrome. Int J Behav Nutr Phys Act.

[13] Beaglehole R, Bonita R, Horton R (2011). Priority actions for the non-communicable disease crisis. Lancet.

[14] Lee IM, Shiroma EJ, Lobelo F, Puska P, Blair SN, Katzmarzyk PT (2012). Effect of physical inactivity on major non-communicable diseases worldwide: and analysis of burden of disease and life expectancy. Lancet.

[15] WHO. Draft comprehensive global monitoring framework and targets for the prevention and control of noncommunicable diseases. Sixty-sixth World Health Assembly. March 15, 2013. Geneva: World Health Organization, 2013.

[16] World Health Organization. Global action plan on physical activity 2018–2030: more active people for a healthier world. https://www.who.int/ncds/prevention/physical-activity/global-action-plan-2018-2030/en/: World Health Organization: Geneva, Switzerland; 2018.

[17] Booth FW, Roberts CK, Laye MJ (2012). Lack of exercise is a major cause of chronic diseases. Compr Physiol.

[18] WHO. Physical activity (2020). Available on https://www.who.int/news-room/fact-sheets/detail/physical-activity, consulted 24th march

[19] Martin SL, Kirkner GJ, Mayo K, Matthews CE, Durstine JL, Hebert JR (2005). Urban, rural, and regional variations in physical activity. J Rural Health.

[20] Bureau Central des Recensements et des Etudes de Population. Rapport de présentation des résultats définitifs du 3ième recensement général de la population au Cameroun; 2010. Available on https://slmp-550104.slc.westdc.net/~stat54/nada/index.php/catalog/89/download/827

[21] Tachang GK, Choukem SP, Ndjebet J, Dzudie A, Titanji VPK (2012). Prevalence of hyperglycaemia, obesity and metabolic syndrome (a three-component study) among hospital personnel in the Littoral region of Cameroon. Int J Med Med Sci.

[22] WHO. Waist Circumference and Waist–Hip Ratio: Report of a WHO Expert Consultation. Geneva: WHO Library Cataloguingin; 2008.

[23] Cleland CL, Hunter RF, Kee F, Cupples ME, Sallis JF, Tully MA (2014). Validity of the global physical activity questionnaire (GPAQ) in assessing levels and change in moderate-vigorous physical activity and sedentary behavior. BMC Public Health.

[24] Glick MR, Ryder KW, Jackson SA (1986). Graphical comparisons of interferences in clinical chemistry instrumentation. Clin Chem.

[25] Bablok W, Passing H, Bender R, Schneider B (1988). A general regression procedure for method transformation. Application of linear regression procedures for method comparison studies in clinical chemistry, Part III. J Clin Chem Clin Biochem..

[26] Friedewald WT, Levy RI, Fredrickson DS (1972). Estimation of the concentration of low-density lipoprotein cholesterol in plasma, without use of the preparative ultracentrifuge. Clin Chem.

[27] Rudovich NN, Rochlitz HJ, Pfeiffer AF (2004). Reduced hepatic insulin extraction in response to gastric inhibitory polypeptide compensates for reduced insulin secretion in normal-weight and normal glucose tolerant first-degree relatives of type 2 diabetic patients. Diabetes.

[28] Ntandou G, Delisle H, Agueh V, Fayomi B (2009). Abdominal obesity explains the positive rural-urban gradient in the prevalence of the metabolic syndrome in Benin. West Africa Nutr Res.

[29] Assah FK, Ekelund U, Brage S, Mbanya JC, Wareham NJ (2011). Urbanization, physical activity, and metabolic health in sub-Saharan Africa. Diabetes Care.

[30] Ntentie FR, Ngondi JL, Azantsa KBG, Santy EV, Dimodi HT, Mbong AM-A, Chakokam NRM, Nguimkeng SB, Zambou H, Oben EJ (2014). Urbanization and metabolic syndrome in Cameroon: alertness on less urbanised areas. Endocrinol Metab Syndr.

[31] Adediran O, Akintunde AA, Edo AE, Opadijo OG, Araoye AM (2012). Impact of urbanization and gender on frequency of metabolic syndrome among native Abuja settlers in Nigeria. J Cardiovasc Dis Res.

[32] Marbou WJT, Kuete V (2019). Prevalence of metabolic syndrome and its components in bamboutos division’s adults, west region of Cameroon. Biomed Res Int.

[33] Bojang KS, Lyrawati D, Sujuti H, Wahono D (2021). Prevalence of metabolic syndrome and its components in Kanifing municipality. The Gambia Med Arch.

[34] Faijer-Westerink HJ, Kengne AP, Meeks KAC, Agyemang C (2019). Prevalence of metabolic syndrome in sub-Saharan Africa: a systematic review and meta-analysis. Nutr Metab Cardiovasc Dis.

[35] Ayina LCN, Sobngwi E, Ngassam E, Ngoa Etoundi LS (2011). Rural and urban differences in metabolic profiles in a Cameroonian population. Pan Afr Med J.

[36] Popkin BM (2001). The nutrition transition and obesity in the developing world. J Nutr.

[37] Hildrum B, Mykletun A, Hole T, Midthjell K, Dahl AA (2007). Age-specific prevalence of the metabolic syndrome defined by the international diabetes federation and the national cholesterol education program: the Norwegian HUNT 2 study. BMC Public Health.

[38] Vieira BA, Luft VC, Schmidt MI, Chambless LE, Chor D, Barreto SM, Duncan BB (2016). Timing and type of alcohol consumption and the metabolic syndrome—ELSA-Brasil. PLoS ONE.

[39] Williamson DF, Madans J, Anda RF, Kleinman JC, Giovino GA, Byers T (1991). Smoking cessation and severity of weight gain in a national cohort. N Engl J Med.

[40] Wilkins JN, Carlson HE, Van Vunakis H, Hill MA, Gritz E, Jarvik ME (1982). Nicotine from cigarette smoking increases circulating levels of cortisol, growth hormone, and prolactin in male chronic smokers. Psychopharmacology.

[41] Fezeu LK, Assah FK, Balkau B, Mbanya DS, Kengne AP, Awah PK, Mbanya JC (2008). Ten-year changes in central obesity and BMI in rural and urban Cameroon. Obesity.

[42] Keller KB, Lemberg L (2003). Obesity and the metabolic syndrome. Am J Crit Care.

[43] Assah F, Mbanya JC, Ekelund U, Wareham N, Brage S (2015). Patterns and correlates of objectively measured free-living physical activity in adults in rural and urban Cameroon. J Epidemiol Community Health.

[44] Mashili FL, Kagaruki GB, Mbatia J, Nanai A, Saguti G, Maongezi S, Magimba A, Mghamba J, Kamugisha M, Mgina E, Mweya CN, Kaushik R, Mayige MT (2018). Physical activity and associated socioeconomic determinants in rural and Urban Tanzania: results from the 2012 WHO-STEPS survey. Int J Popul Res.

[45] Mbalilaki JA, Hellenius ML, Masesa Z, Hostmark AT, Sundquist J, Stromme SB (2007). Physical activity and blood lipids in rural and urban Tanzanians. Nutr Metab Cardiovasc Dis.

[46] Weng X, Liu Y, Ma J, Wang W, Yang G, Caballero B (2007). An urban-rural comparison of the prevalence of the metabolic syndrome in Eastern China. Public Health Nutr.

[47] Chiwanga FS, Njelekela MA, Diamond MB, Bajunirwe F, Guwatudde D, Nankya-Mutyoba J, Kalyesubula R, Adebamowo C, Ajayi I, Reid TG, Volmink J, Laurence C, Adami HO, Holmes MD, Dalal S (2016). Urban and rural prevalence of diabetes and pre-diabetes and risk factors associated with diabetes in Tanzania and Uganda. Glob Health Action.

[48] Guwatudde D, Nankya-Mutyoba J, Kalyesubula R, Laurence C, Adebamowo C, Ajayi I, Bajunirwe F, Njelekela M, Chiwanga FS, Reid T, Volmink J, Adami HO, Holmes MD, Dalal S (2015). The burden of hypertension in sub-Saharan Africa: a four-country cross sectional study. BMC Public Health.

[49] van de Vijver S, Akinyi H, Oti S, Olajide A, Agyemang C, Aboderin I, Kyobutungi C (2013). Status report on hypertension in Africa–consultative review for the 6th session of the African union conference of ministers of health on NCD’s. Pan Afr Med J.

[50] Wayas FA, Smith JA, Lambert EV, Guthrie-Dixon N, Wasnyo Y, West S, Oni T, Foley L (2023). Association of perceived neighbourhood walkability with self-reported physical activity and body mass index in South African adolescents. Int J Environ Res Public Health.

[51] Thyfault JP, Bergouignan A (2020). Exercise and metabolic health: beyond skeletal muscle. Diabetologia.

[52] Owen CG, Nightingale CM, Rudnicka AR, Sattar N, Cook DG, Ekelund U, Whincup PH (2010). Physical activity, obesity and cardiometabolic risk factors in 9- to 10-year-old UK children of white European, South Asian and black African-Caribbean origin: the Child Heart And health Study in England (CHASE). Diabetologia.

[53] Bremer AA, Auinger P, Byrd RS (2009). Relationship between insulin resistance-associated metabolic parameters and anthropometric measurements with sugar-sweetened beverage intake and physical activity levels in US adolescents: findings from the 1999–2004 national health and nutrition examination survey. Arch Pediatr Adolesc Med.

[54] Laaksonen DE, Lakka HM, Salonen JT, Niskanen LK, Rauramaa R, Lakka TA (2002). Low levels of leisure-time physical activity and cardiorespiratory fitness predict development of the metabolic syndrome. Diabetes Care.

[55] Ekelund U, Griffin SJ, Wareham NJ (2007). Physical activity and metabolic risk in individuals with a family history of type 2 diabetes. Diabetes Care.

[56] Lee J, Kim Y, Jeon JY (2016). Association between physical activity and the prevalence of metabolic syndrome: from the Korean National Health and Nutrition Examination Survey, 1999–2012. Springerplus.

[57] Powers SK, Hamilton K (1999). Antioxidants and exercise. Clin Sports Med.

[58] Green JS, Stanforth PR, Rankinen T, Leon AS, Roa DC, Skinner JS, Bouchard C, Wilmore JH (2004). The effects of exercise training on abdominal visceral fat, body composition, and indicators of the metabolic syndrome in postmenopausal women with and without estrogen replacement therapy: the HERITAGE family study. Metabolism.

[59] Kasapis C, Thompson PD (2005). The effects of physical activity on serum C-reactive protein and inflammatory markers: a systematic review. J Am Coll Cardiol.

